# Shortening treatment duration for uncomplicated community acquired pneumonia to align with best practice guidelines in a large academic paediatric emergency department

**DOI:** 10.1016/j.fhj.2024.100142

**Published:** 2024-05-07

**Authors:** Ryan E.St. Pierre-Hetz, Brielle Stanton Skotnicki, Christine R. Aspiotes, Brett McAninch, Johanna R. Rosen

**Affiliations:** a University of Pittsburgh, UPMC Children's Hospital of Pittsburgh, United States; b UPMC Children's Hospital of Pittsburgh, United States

## Abstract

Recommendations for antibiotic therapy for community acquired pneumonia recently changed from 10-day to 5-day duration. This quality improvement project aimed to change the practice of providers in an urban, free-standing children's hospital paediatric emergency department in the United States to match the updated recommendations. Improvement interventions included educational outreach, data sharing, on-site reminders, and audit-and-feedback. The project included analysis of ED return visits to monitor possible treatment failure. Interventions successfully increased 5-day antibiotic prescription rate from 3% to 85% within 12 months.

## Introduction

Antibiotic stewardship has become increasingly important over the last decade given the emergence of drug resistance. Until recently, the best treatment for uncomplicated community acquired pneumonia (CAP) in the paediatric population was thought to be a 10-day course of high-dose amoxicillin; however, multiple recent studies challenge this, showing similar cure rates with a 5-day course of amoxicillin.^1^, ^2^, ^3^, ^4^ By reducing the course of amoxicillin, there is potential for reduced side effects (ie diarrhoea, nausea),^5^ improved cost and adherence. At project initiation, only 3% of our paediatric emergency department (PED) providers prescribed a 5-day course of amoxicillin for uncomplicated CAP. We aimed to increase this rate to 85%.

Our team was composed of PED clinicians supported by paediatric infectious disease experts. We used the Institute for Healthcare Improvement's Model for Improvement to create a specific goal and test interventions like educational outreach, data sharing, on-site reminders, audit and feedback.^6^

Smart aim: Improve adherence to best practice guidelines for uncomplicated CAP for patients from 6 months to 17 years old who are discharged from the PED. Specifically, our goal was to improve PED prescribing practice from the baseline of 3% to 85% for total duration of 5 days of amoxicillin, over a 12-month period.

## Methods

### Initial education

Our focus was education of providers in the PED at our free-standing children's hospital in the United States. The project was presented monthly at provider staff meetings from June 2022 to August 2023 including a review of literature, our smart aim, baseline data and an updated run-chart trending prescription practice. In the last 3 months, we included audit and feedback slides championing those prescribing the shorter course of amoxicillin. Additionally, institutional guidelines were updated with these best practice recommendations. These guidelines are frequently employed by providers throughout the hospital for up-to-date practice standards. Lastly, our team met with local primary care provider (PCP) groups to share our practice change.

### Maintenance

Just-in-time education was provided to reinforce evidence-based prescribing practices. Multiple reminder cards were placed on workstations throughout the PED to remind providers of the shorter duration of antibiotics for CAP.

Chart audits were performed for all patients diagnosed with CAP and discharged from the PED. Directed feedback was given to any prescriber that provided over 5 days of antibiotics to patients with CAP.

Lastly, our team updated our provider groups with run charts, successes, and areas for improvement. This allowed for re-education and the ability to resolve any issues that our providers encountered in real time.

## Results

**Fig. 1:**
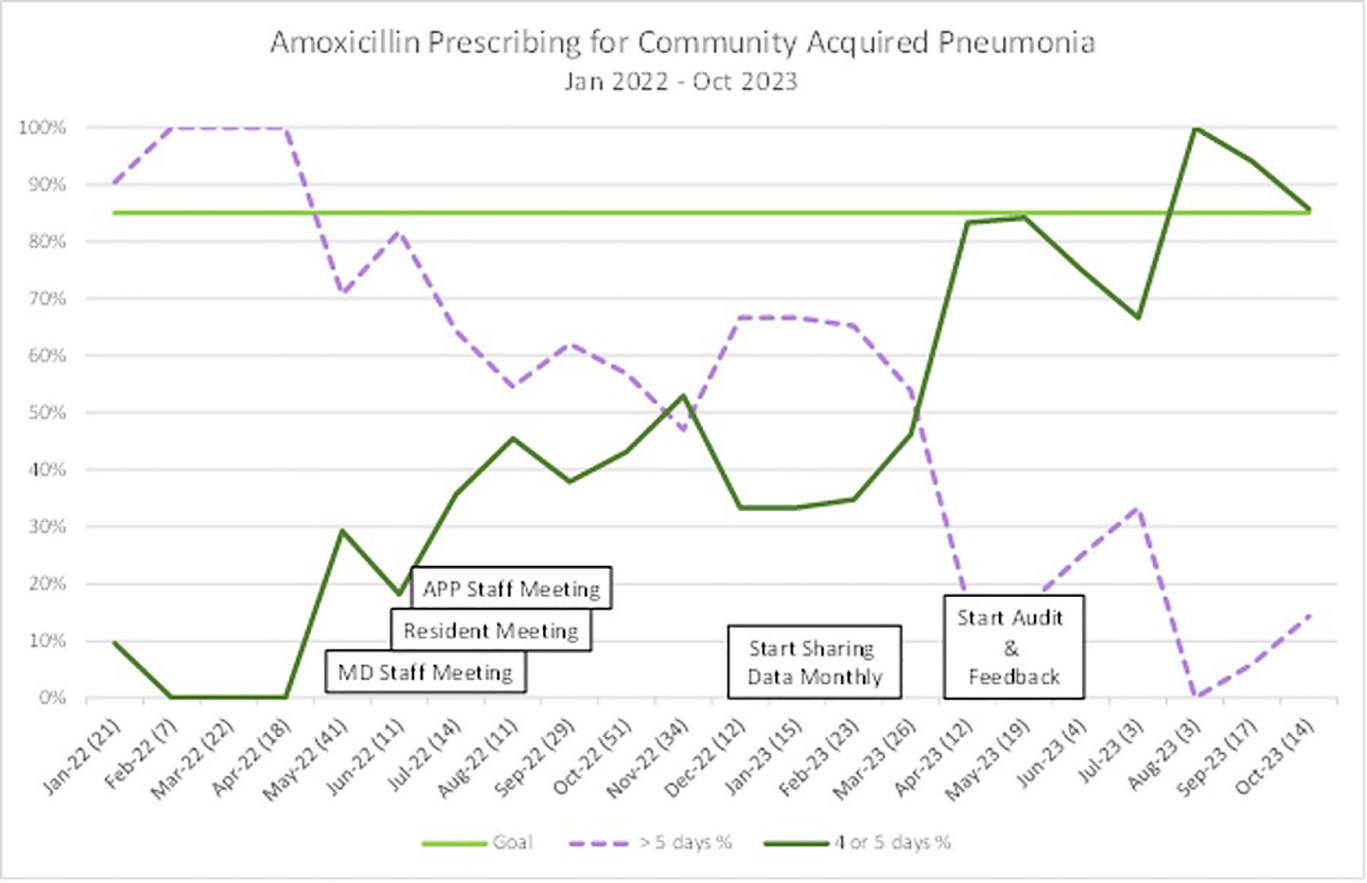
Run chart.

Our outcome measure was percentage of amoxicillin prescriptions that were between 4 and 5 days out of total prescriptions for CAP. This measure was queried from the medical records. Fig. 1 shows our rate of 4–5-day prescriptions, that reached goal within 6 months of project implementation. There was a decrease below goal that was addressed with our audit and feedback process, returning our 4–5 day prescription rate back above our goal of 85%.

We selected ED return visits within 7 days as our balancing measure, to review for possible treatment failure. We performed manual chart reviews for return visit and invited a new team member to adjudicate the cases to limit bias. Three children were prescribed 5 days of amoxicillin and returned to the ED within 7 days, but none of these cases represented clear treatment failure. One child re-presented 4 days after the initial visit and was triaged to an urgent care. Urgent care providers obtained a chest radiograph which was negative for pneumonia. The patient was treated with intramuscular ceftriaxone, prescribed an additional 5 days of amoxicillin and discharged home. Urgent care practitioners were not part of the target change group during this phase of the improvement project and may not have been aware of the new practice for shorter course therapy. A second child returned 3 days after the initial ED visit for fussiness, cough and decreased oral intake. This child left the waiting room prior to being seen by a provider. The patient's PCP was outside of our medical system, but there were no return visits to the PED. A third child returned 3 days after the initial ED visit. On review, the patient had autism and was unable to consistently take his amoxicillin. He did not experience treatment failure and was discharged home.

## Discussion

With a small team, we used simple educational tools with no monetary cost and low time cost to change practices in a large PED. These interventions were successful within a year of implementation. Education, audit and involvement of key participants were effective in changing practice.

Our project demonstrates how impactful targeted education, personalised feedback and data-sharing can be, even for a busy academic PED. Further efforts may include spread to community EDs and urgent care settings and could include electronic health record decision support.

The limitations of our study are that it is a single-centre project limited to the PED. While our PED sees nearly 80,000 patients a year, involving multiple centres would increase the strength of our findings. Additionally, we were unable to access charts for centres outside our medical system. Thus, it is difficult to obtain information on CAP treatment failure that was managed outside of our system.

## Conclusion

This project demonstrates how a small team of providers employing low-cost targeted interventions were successful in changing the practice of a large and high-volume academic PED. It would be easily reproduced in any centre and would not require significant financial burden or specialised intervention.

## Declaration of competing interest

The authors declare that they have no known competing financial interests or personal relationships that could have appeared to influence the work reported in this paper.

## Appendix Supplementary materials

Image, application 1
