# Supplementary figures and images for: Shortening treatment duration for uncomplicated community acquired pneumonia to align with best practice guidelines in a large academic paediatric emergency department
Source: Future Healthc J. 2024 May 7;11(2):100142. doi: 10.1016/j.fhj.2024.100142 (PMC11126758; doi:10.1016/j.fhj.2024.100142)

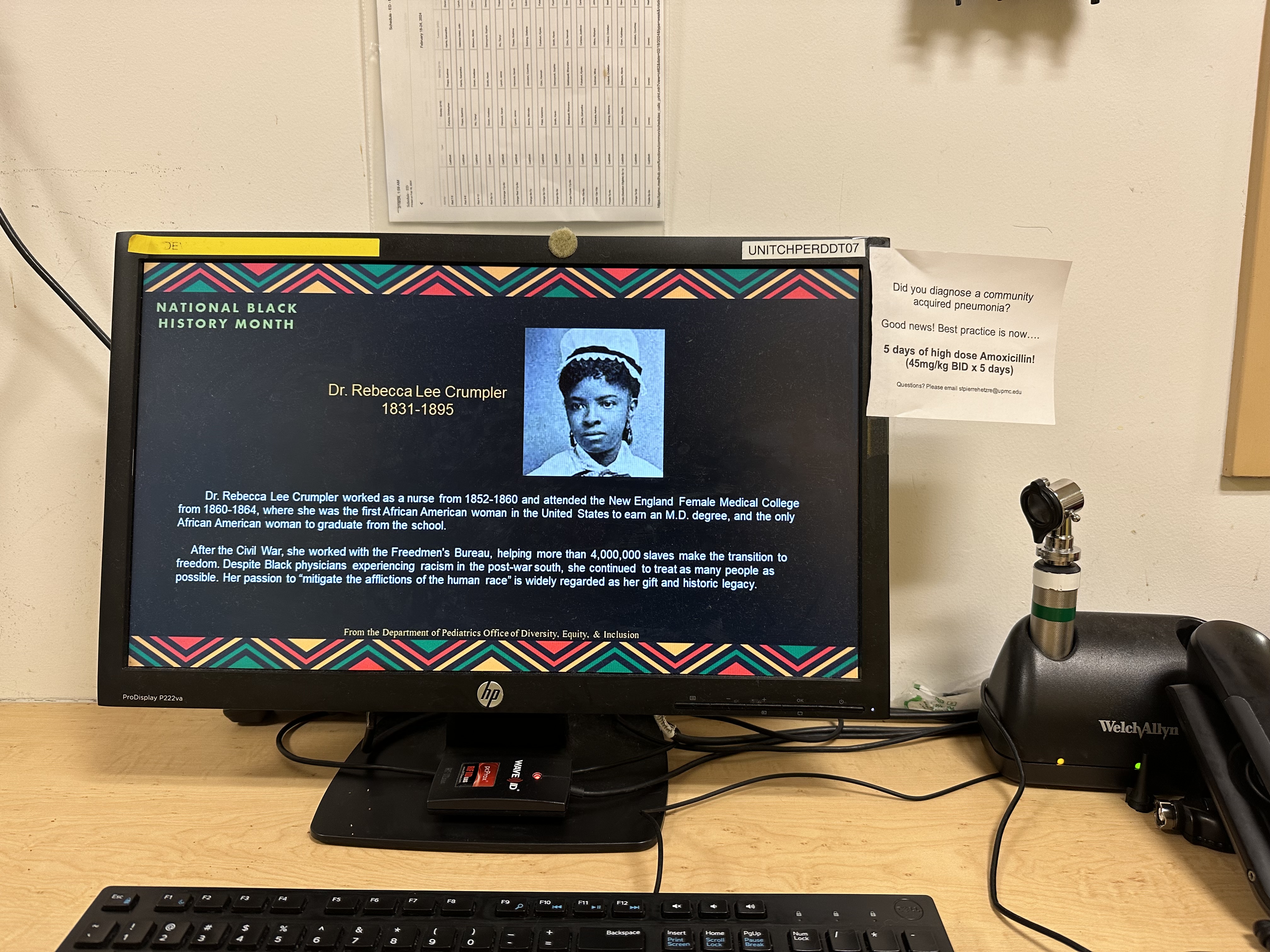
Workstation provider reminder cards
Source: Johanna Rosen, 2024

Supplement: Supplementary file 1 [file mmc1.docx]
